# Knowledge, Attitudes, and Practices of Adult Residents of Riyadh Regarding Sunstrokes

**DOI:** 10.7759/cureus.75306

**Published:** 2024-12-08

**Authors:** Fahad A Alfrayan, Mohammed A Alkathiri, Basel A Fakeeha, Abdulrahman A Alfaifi, Yusuf M Alkahtani, Ahmad M Alkhayatt, Mohammad Y Abdulghani, Bader M Altamimi, Ali Al-Hazmi

**Affiliations:** 1 College of Medicine, King Saud University, Riyadh, SAU

**Keywords:** attitude, cross-sectional study, kap, knowledge, practice, preferred channel, riyadh city, sunstroke

## Abstract

Objectives

This study aims to evaluate the knowledge, attitudes, and practices of adult residents in the city of Riyadh regarding sunstrokes and identify the preferred channel for spreading awareness about sunstrokes among this population.

Methods

This study employed an analytical cross-sectional design targeting Riyadh city residents aged 18 and older. A multistage random sampling technique was used, resulting in a sample size of 681 participants. Data were collected through an online questionnaire that included demographic information, knowledge, attitudes, and practices related to sunstrokes and preferred methods for obtaining information about sunstrokes. Statistical analysis was performed using IBM SPSS Statistics for Windows, Version 21 (Released 2012; IBM Corp., Armonk, New York).

Results

Out of 681 participants, nearly half demonstrated good knowledge about sunstrokes (48.8%). The majority exhibited excellent attitudes (85.5%), and two-thirds showed outstanding practices (68.6%). Attitude and practice scores were significantly associated with age, with higher mean ranks observed among older participants and a decline in scores in younger age groups. Regarding social status, widows had the highest mean ranks in knowledge (516.5) and practice (420), while married participants scored highest in attitude (387.85).

Conclusion

The study revealed that the participants had generally good knowledge about sunstrokes, and their attitudes and practices were notably high. The most frequently utilized sources of information were electronic platforms.

## Introduction

According to an ongoing temperature analysis conducted by scientists at the National Aeronautics and Space Administration Goddard Institute for Space Studies, the average temperature on Earth has increased by a little more than 1°C (2°F) since 1880. Two-thirds of the warming has occurred since 1975, at a rate of roughly 0.15-0.20°C per decade [1]. Consequently, heat waves are expected to become more frequent, more intense, and longer [2]. The impacts will be more intense across the Middle East, of which Saudi Arabia is a part, and North Africa, a region mostly characterized by a hot and arid climate, which is already intolerable for human beings in many parts [3].

Heat waves can result in deaths, such as the heat wave that hit France in 2003, which resulted in more than 14,800 deaths between August 1 and 20 [4], and heat-related illnesses (HRI), one of which is sunstroke (or heatstroke) [5]. Sunstroke is a potentially life-threatening acute syndrome characterized, traditionally, by a core body temperature >40.6°C (105.1°F) and severe neurologic symptoms, including confusion and seizures, along with disorientation and a lack of sweating [5-7]. In addition to exposure to hot weather, the risk factors for heatstroke include physiological factors illustrated by cardiovascular insufficiency, inadequate peripheral vasodilation, reduction in capillary density and quality of cutaneous microcirculation, and decreased sweat rate and sweat gland outputs [8].

Sunstroke has a reported global mortality rate of approximately 12% in adult cases worldwide [9]. Data from Saudi Arabia show a seasonal incidence pattern ranging from 22 to 250 cases per 100,000 capita, and the crude mortality rate in Saudi Arabia has been estimated at 50% [10]. Despite the understanding of the pathophysiology of HRIs, pharmacological interventions are limited. Prevention is the best strategy against HRI [5].

The three key aspects of knowledge, attitudes, and practices (KAP) toward sunstrokes reduce the adverse health impacts of heat waves [11]. To the best of our knowledge, limited studies have explored KAP related to sunstroke in Saudi Arabia, with most focusing on the Hajj season in Mecca. However, Riyadh presents a distinct context due to its extreme summer temperatures, rapid urbanization, and different population dynamics. Despite these factors, to our knowledge, no studies to date have specifically investigated sunstroke-related KAP among Riyadh residents. This study addresses this gap by providing valuable insights into local public health needs and identifying areas for targeted interventions to mitigate the health impacts of sunstroke in this unique setting; therefore, the research question is, "What are the knowledge, attitudes, and practices of adult residents of Riyadh regarding sunstrokes in 2021?" The study's objectives are (1) to assess the general KAP of Riyadh's adult residents about sunstroke and (2) to recognize the preferred channel for spreading awareness of sunstroke among Riyadh's adult residents. Our findings will contribute to the understanding of the public's KAP with regard to sunstrokes and their preferred channel, which, in turn, will assist local health authorities with plans and implementation for coping with sunstrokes and the corresponding adverse effects.

## Materials and methods

Study design

This analytical cross-sectional study was conducted over six months, from June to December 2021, in various malls across the city of Riyadh. The study targeted adult residents of Riyadh, with the inclusion criteria set as residents aged 18 years or older who could read Arabic. The exclusion criteria were non-residents, individuals under 18, and non-Arabic readers.

The sampling technique used was multistage random sampling. Riyadh was divided into five geographical regions: north, south, east, west, and center. The number of malls in each area was identified, and one mall from each region was randomly selected. The selected malls were AlSalam Mall (west), Khurais Mall (east), Mode Mall (central), Riyadh Front (north), and Riyadh Avenue (south). Data collection occurred from October 1 to 24, 2021, between 4:00 PM and 10:00 PM.

Sample size

The estimated sample size was 473 participants, calculated with a margin of error of 3.5% and a confidence level of 95% based on a sample proportion of 81.5% [12]. The calculation followed the equation:

$N=\frac{1.96^{2}0.815(1-0.815)}{0.035^{2}}$

To account for potential nonresponses, an additional 20% was added, bringing the minimum required sample size to 568 participants. After excluding those who did not meet the inclusion criteria, the final sample size was 681 participants.

Ethical considerations

Ethical approval for this study was obtained from the Institutional Review Board of King Saud University College of Medicine (No. E-21-6146). Informed consent was integrated into the survey questionnaire, which clearly explained the purpose of the study and the participant's right to withdraw at any time without any obligation to the research team. Anonymity was ensured by not collecting names or personal identifiers. Additionally, the respondents were informed that no incentives or rewards would be provided for participation.

Data collection/data source

Data were collected in the selected malls through QR codes given to the participants, which directed them to an online questionnaire in Arabic. The questionnaire was designed by reviewing available surveys in the literature [11-15] and consisted of four sections. The first collected demographic information, such as age, gender, and education level. The second assessed the participants' knowledge about sunstrokes, and the third focused on attitudes and practices related to sunstroke prevention. The fourth section inquired about the preferred channels for receiving information on sunstrokes.

Statistical analysis

For the statistical analysis, a previously developed KAP scoring system was adapted from prior research and used [12]. Incorrect, inappropriate, or uncertain responses were scored as 0, while correct or appropriate responses received a score of 1. For the multiple-choice questions with more than one correct answer, a score of 1 was given for each correct response, while incorrect answers received a score of 0. The overall mean scores for each element of KAP ranged from 0 to 1. These scores were further divided into four categories to reflect the participants' levels of KAP: very poor (score<0.25), poor (0.25≤score<0.50), good (0.5≤score< 0.75), and excellent (score≥0.75).

The data were analyzed using IBM SPSS Statistics for Windows, Version 21 (Released 2012; IBM Corp., Armonk, New York). Descriptive statistics, including frequencies, percentages, and means, were used to summarize the categorical and quantitative variables. The Kruskal-Wallis test was employed to compare the mean ranks of the quantitative outcome variables across different categorical variables. Statistical significance was defined as a p-value ≤0.05, and 95% confidence intervals were used to report the precision of the results.

## Results

The 681 survey participants' characteristics and sources of information about sunstroke are presented in Table 1. Among the participants, 324 (47.6%) were male, and 349 (51.2%) were private sector employees working in the malls. The participants' overall health status was good, although 29 (4.3%) had diabetes, 36 (5.3%) had hypertension, 43 (6.3%) had asthma, and 86 (12.6%) were obese. The participants selected websites (77.2%) and social media (72.1%) as their preferred sources of information about sunstroke.

**Table 1:** Sociodemographic and comorbidity characteristics of the survey participants and their sources of information about sunstroke (N=681)

Variable	N (%)
Geographical location	
Alsalam Mall (west)	138 (20.3)
Khurais Mall (east)	144 (21.1)
Riyadh Front (north)	144 (21.1)
Riyadh Avenue Mall (south)	103 (15.1)
Mode Mall (center)	152 (22.3)
Gender	
Male	324 (47.6)
Female	357 (52.4)
Age	
18-24	219 (32.2)
25-34	259 (38.0)
35-44	137 (20.1)
45 and above	66 (9.7)
Education level	
Elementary	4 (0.6)
Middle school	20 (2.9)
High school	171 (25.1)
Bachelor's degree	435 (63.9)
Master's degree or higher	51 (7.5)
Occupation	
Student	95 (14)
Government sector employee	144 (21.1)
Private sector employee	349 (51.2)
Retired	18 (2.6)
Unemployed	75 (11.0)
Social status	
Single	358 (52.6)
Married	301 (44.2)
Divorced	20 (2.9)
Widow	2 (0.3)
Family monthly income (in Saudi Riyal)	
Less than 5,000	203 (29.8)
5,000-9,999	153 (22.5)
10,000-14,999	81 (11.9)
15,000-19,999	51 (7.5)
20,000 and above	58 (8.5)
I do not know	37 (5.4)
I do not want to answer	98 (14.4)
Comorbidity	
Diabetes (yes)	29 (4.3)
Hypertension (yes)	36 (5.3)
Asthma (yes)	43 (6.3)
Obesity (yes)	86 (12.6)
Sources of information about sunstroke	
Physicians (yes)	459 (67.4)
Family and friends (yes)	458 (67.3)
Social media (yes)	491 (72.1)
Websites (yes)	526 (77.2)
Newspapers/magazines (yes)	218 (32.0)
Radio (yes)	273 (40.1)
Television (yes)	386 (56.7)

Almost half of the participants had good knowledge about sunstroke (48.8%), and more than a quarter had excellent knowledge (29.8%). Only two of the knowledge items received appropriate responses from more than 75% of the participants. Most respondents (86%) were aware that drinking fluids and using cold water helps control sunstroke, and two-thirds (64.9%) were aware that sweating can lower body temperature. By contrast, less than one-fifth (17.9%) were aware that sunstrokes can occur during sleep (Table 2).

**Table 2:** Distribution of the survey participants' responses to the sunstroke knowledge items

Item	N (%)
Heatstroke is common in Saudi Arabia.	
Strongly agree	241 (35.4)
Agree	258 (37.9)
Neutral	131 (19.2)
Disagree	47 (6.9)
Strongly disagree	4 (0.6)
Heatstroke can be fatal.	
Strongly agree	210 (30.8)
Agree	254 (37.3)
Neutral	139 (20.4)
Disagree	71 (10.4)
Strongly disagree	7 (1.0)
If I wear light-colored clothes, I will feel cool in summer.	
Strongly agree	130 (19.1)
Agree	261 (38.3)
Neutral	143 (21.0)
Disagree	121 (17.8)
Strongly disagree	26 (3.8)
Fever, fatigue, and shortness of breath are common symptoms of heatstroke.	
Strongly agree	133 (19.5)
Agree	286 (42.0)
Neutral	185 (27.2)
Disagree	67 (9.8)
Strongly disagree	10 (1.5)
Some medicines increase the risk of heatstroke.	
Strongly agree	71 (10.4)
Agree	160 (23.5)
Neutral	285 (41.9)
Disagree	140 (20.6)
Strongly disagree	25 (3.7)
Green plants play a role in cooling.	
Strongly agree	371 (54.5)
Agree	235 (34.5)
Neutral	58 (8.5)
Disagree	15 (2.2)
Strongly disagree	2 (0.3)
Drinking fluids and using cold water helps control sunstroke.	
Strongly agree	323 (47.4)
Agree	263 (38.6)
Neutral	67 (9.8)
Disagree	22 (3.2)
Strongly disagree	6 (0.9)
Sweating can lower body temperature.	
Strongly agree	182 (26.7)
Agree	260 (38.2)
Neutral	163 (23.9)
Disagree	61 (9.0)
Strongly disagree	15 (2.2)
Elderly people are more susceptible to heatstroke.	
Strongly agree	180 (26.4)
Agree	215 (31.6)
Neutral	200 (29.4)
Disagree	73 (10.7)
Strongly disagree	13 (1.9)
Heat cramps and heat stress can precede heatstroke.	
Strongly agree	116 (17.0)
Agree	269 (39.5)
Neutral	244 (35.8)
Disagree	44 (6.5)
Strongly disagree	8 (1.2)
Being thirsty is the only sign of needing to drink water.	
Strongly agree	133 (19.5)
Agree	146 (21.4)
Neutral	82 (12.0)
Disagree	237 (34.8)
Strongly disagree	83 (12.2)
Sunstrokes can occur during sleep.	
Strongly agree	37 (5.4)
Agree	85 (12.5)
Neutral	182 (26.7)
Disagree	236 (34.7)
Strongly disagree	141 (20.7)
Knowledge range	
Excellent knowledge	203 (29.8)
Good knowledge	332 (48.8)
Poor knowledge	123 (18.1)
Very poor knowledge	23 (3.4)

Most of the participants had excellent attitude scores (85.5%), and two-thirds had excellent practice scores (68.6%). Three-quarters of the attitude items received appropriate responses from more than 80% of those surveyed, with 90.3% willing to take sunstroke prevention measures whenever a temperature warning was issued. As for practices, 87.3% of the participants stated that they used a fan or air conditioning to reduce body temperature. Additionally, over 80% intended to take sunstroke prevention measures when the temperature was high. However, only 62.4% reported checking the weather forecast before going out (Table 3).

**Table 3:** Distribution of the survey participants' responses to the sunstroke-related attitudes and practices items

Items	N (%)
Attitude	
During a hot day, I consume more water even when I do not feel thirsty. (yes)	610 (89.6)
I intend to take sunstroke prevention measures if a temperature warning is issued. (yes)	615 (90.3)
If the weather is extremely hot, I postpone performing chores until it becomes cooler. (yes)	547 (80.3)
I prefer drinking soft drinks when I feel thirsty. (no)	479 (70.3)
Attitude range	
Excellent attitude	582 (85.5)
Good attitude	79 (11.6)
Poor attitude	16 (2.3)
Very poor attitude	4 (0.6)
Practice	
I intend to take sunstroke prevention measures when the temperature is high.	
Strongly agree	334 (49.0)
Agree	244 (35.8)
Neutral	83 (12.2)
Disagree	15 (2.2)
Strongly disagree	5 (0.7)
I always drink water and fluids even when I am not thirsty.	
Strongly agree	327 (48.0)
Agree	248 (36.4)
Neutral	74 (10.9)
Disagree	29 (4.3)
Strongly disagree	3 (0.4)
I arrange outdoor activities only at cooler times.	
Strongly agree	241 (35.4)
Agree	246 (36.1)
Neutral	122 (17.9)
Disagree	60 (8.8)
Strongly disagree	12 (1.8)
I stop exercising when I have heat cramps or heat stress.	
Strongly agree	270 (39.6)
Agree	267 (39.2)
Neutral	92 (13.5)
Disagree	39 (5.7)
Strongly disagree	13 (1.9)
I check the weather forecast before going out.	
Strongly agree	212 (31.1)
Agree	213 (31.3)
Neutral	136 (20.0)
Disagree	90 (13.2)
Strongly disagree	30 (4.4)
I use sunscreen when the temperature is high.	
Strongly agree	246 (36.1)
Agree	191 (28.0)
Neutral	107 (15.7)
Disagree	84 (12.3)
Strongly disagree	53 (7.8)
I dress based on the weather conditions.	
Strongly agree	327 (48)
Agree	256 (37.6)
Neutral	66 (9.7)
Disagree	21 (3.1)
Strongly disagree	11 (1.6)
I use a fan or air conditioning to reduce body temperature.	
Strongly agree	385 (56.5)
Agree	210 (30.8)
Neutral	56 (8.2)
Disagree	21 (3.1)
Strongly disagree	9 (1.3)
Practice range	
Excellent practice	467 (68.6)
Good practice	146 (21.4)
Poor practice	54 (7.9)
Very poor practice	14 (2.1)

Table 4 illustrates the mean comparison of the KAP scores across the survey participants' sociodemographic characteristics. No significant association was found for the three KAP elements relative to geographical location. However, for gender, the practices mean rank score was significant, with females higher than males (370.1 and 308.94, respectively). Relative to age, the attitudes and practices scores were significant, with their mean ranks higher in the older age group and descending as the age groups became younger. Attitudes and practices were also significant relative to occupation, as the retired mean rank was the highest (416.83 for attitudes and 388.31 for practices). Educational level was only significant in the knowledge score, with master's degree and higher having the highest mean rank (429.46). Regarding social status, widows had the highest mean rank scores for knowledge (516.5) and practices (420), while married people had the highest mean rank score for attitudes (387.85). The knowledge and attitudes scores were significant relative to family monthly income, with the knowledge mean rank being the highest in the 5,000-9,999 group, followed by 20,000 and above. The attitudes mean rank was greatest for 15,000-19,999, followed by 20,000 and above.

**Table 4:** Comparison of the survey participants' sunstroke knowledge, attitudes, and practices scores in relation to their characteristics *P-value from Kruskal-Wallis test **P < 0.05

Variable	Knowledge Score Mean Rank	Knowledge Chi-Square (X^2^)	Knowledge Score p-value*	Attitudes Score Mean Rank	Attitudes Chi-Square (X^2^)	Attitudes Score p-value*	Practices Score Mean Rank	Practices Chi-Square (X^2^)	Practices Score p-value*
Geographical location									
Alsalam Mall (west)	354.21	8.353	0.079	362.48	4.198	0.380	379.39	9.331	0.053
Khurais Mall (east)	313.81			323.21			325.42		
Riyadh Front (north)	333.84			350.67			316.17		
Riyadh Avenue Mall (south)	324.66			333.12			352.41		
Mode Mall (center)	372.62			334.53			336.70		
Gender									
Male	335.29	0.529	0.467	342.04	0.021	0.885	308.94	17.264	<0.0001**
Female	346.18			340.05			370.10		
Age									
18–24	313.82	0.6292	0.098	279.68	47.495	<0.0001**	291.65	37.135	<0.0001**
25–34	332.58			333.56			321.59		
35–44	358.65			390.97			395.62		
45 and above	366.89			419.61			416.67		
Educational level									
Elementary	305.00	15.669	0.003**	256.50	8.093	0.088	426.13	1.354	0.852
Middle school	387.75			306.03			370.33		
High school	311.65			330.51			336.71		
Bachelor’s degree	340.35			340.37			340.29		
Master’s degree or higher	429.46			401.89			343.26		
Occupation									
Student	302.63	4.787	0.310	293.96	21.591	<0.0001**	295.43	12.407	0.0 15*
Government sector employee	350.79			388.63			371.93		
Private sector employee	348.79			328.73			332.37		
Retired	321.56			416.83			388.31		
Unemployed	339.21			348.02			368.15		
Social status									
Single	322.86	9.267	0.026**	302.46	37.195	<0.0001**	308.04	22.714	<0.0001**
Married	363.52			387.85			375.66		
Divorced	309.25			331.20			401.43		
Widow	516.50			288.00			420.00		
Family monthly income									
Less than 5,000	344.80	12.566	0.050**	308.45	23.614	<0.0001**	341.19	9.111	0.167
5,000–9,999	364.40			358.15			362.42		
10,000–14,999	340.69			360.67			349.36		
15,000–19,999	356.55			413.29			351.00		
20,000 and above	362.55			382.71			356.91		
I do not know	262.16			309.76			280.27		
I do not want to answer	305.78			314.89			308.56		

## Discussion

Studies have found that extremely high temperatures have negative effects on human health [16,17]. However, few studies have explored the public's KAP about heat waves in Saudi Arabia, and most of them have focused on the Hajj season. Research on heat wave-related KAP in Saudi Arabia may be significant, as the kingdom is severely affected by heat events. The findings of such studies may encourage the Ministry of Health to increase its efforts in educating people about sunstrokes and address the effects and risks of global or regional heat wave events [18,19].

To our knowledge, this is the first study to investigate the public's KAP about heat waves in the Riyadh region. We found that websites (77.2%) and physicians (67.4%) were the most used and third most used sources of information, respectively, which is somewhat consistent with Alduraywish et al.'s findings [20], where physicians and pharmacists were the most used sources of information, followed by internet searches. Social media was the least preferred choice in Alduraywish et al.'s study, whereas social media came in second place (72.1%) in ours. This may be because Alduraywish et al.'s study was conducted before the COVID-19 pandemic, which had an impact on people's contact with physicians during lockdown. According to Ho et al. [21], people became more reliant on social media and internet searches in this period.

Our survey revealed that 48% of Riyadh's residents generally had good knowledge about sunstroke, which was lower than 80% with excellent knowledge scores in a study conducted about Hajj, as pilgrims receive the education necessary to handle the heat during Hajj. Among our participants, 55% believed that heatstroke cannot occur during sleep, compared to 84% in Japan [15]. This may be due to Riyadh's hot climate, with people rarely sleeping outside when the sun is out and most sleeping with air conditioning.

Most Riyadh residents exhibited excellent practices (68.6%) and attitudes (85.5%), which was supported by two studies in China that reported behavioral alterations for the majority of their participants [11,22]. Yezli et al. indicated that 89% declared they dressed based on weather conditions, but only 43% checked the weather forecast before going out, corroborating our results [12]. Li et al. found that 80% implemented sunstroke prevention, and 88.6% arranged outdoor activities at cooler times [11]. Our results showed that 84.8% followed preventive measures, and 71.5% arranged outdoor activities at cooler times, suggesting that preventive measures were taken almost equally, but a lower percentage of participants arranged activities in cooler weather [11]. Contrary to 52% of pilgrims who only drank water when thirsty during Hajj [12], 84.4% of our respondents reported drinking water even when not thirsty. Air conditioning, while not the most effective method for reducing body temperature during heatstroke, is widely accessible and commonly used as an initial intervention in cases of classic heatstroke [8]. Its increased use as a prevention measure has also been associated with reduced or no HRI mortality in the United States [5]. Conversely, a study in China reported that only 6.3% of respondents sought air-conditioned locations to cope with heat [22]. In Riyadh, however, the majority of residents (87.3%) reported using a fan or air conditioning to manage heat exposure, highlighting a stronger reliance on these measures for both prevention and relief of heat-related stress.

In this study, female participants had significantly better practices regarding sunstroke than male participants. Supporting our findings, a study found that among the public in China, females had better practices regarding heat waves, although they had lower knowledge scores [11]. The authors of that study reasoned that the difference between genders was due to the greater impact of heat on women compared to men. We found that having a master's degree or higher education correlated with significantly better knowledge regarding sunstroke; multiple studies have reported similar observations [11,15,23]. For instance, holders of master's degrees or higher were the most knowledgeable about heat waves in China. By contrast, a study conducted in Lisbon and Madrid showed that educational level was not significantly associated with lower knowledge [13]. The authors of that study suggested that this may be due to the frequent exposure of citizens in these cities to extreme heat, which might reduce the influence of education on awareness. We also found that older age was significantly associated with better attitudes and practices relating to sunstroke. This is significant given that elderly people are more vulnerable to sunstrokes and consequently have a higher risk of dying [24-26]. A study among Arab pilgrims reported those over 40 years old had better health-related practices, including greater use of head covers and drinking more water [27].

The present study has some limitations. First, some groups had few participants, notably the elderly, individuals with comorbidities, and those at the extremes of educational levels, which may have affected our results. Moreover, social desirability bias may have been a factor, as some participants could have provided answers that they believed were in favor of the survey. Finally, some malls refused to cooperate, which prolonged the data-collecting period. Despite these limitations, our findings may be helpful to the Ministry of Health, which we encourage to pay close attention to the protection of the population, particularly vulnerable groups, and to increase their knowledge. We recommend focusing on websites, as the majority prefer them to gain heat-related knowledge to prevent heat illnesses, as shown in the results.

## Conclusions

Residents of Riyadh have above-average knowledge and comparatively high attitudes and habits regarding sunstroke. Nonetheless, our research revealed particular information gaps that can guide focused efforts. For example, vulnerable groups, such as the elderly, those with comorbidities, and those with lower incomes and educational levels, need specialized health education programs to increase their knowledge of the dangers of sunstroke and how to prevent it.

Diverse demographics could be effectively reached using electronic media as the main medium for spreading health information connected to heat. The government should also implement thorough risk-awareness programs, including community-based interventions and educational programs, to raise awareness of the negative impacts of heat and encourage flexible coping mechanisms. Lastly, additional large-scale research is required to assess the efficacy of adopted interventions and to offer deeper insights into behaviors related to sunstroke, especially those that employ qualitative methodologies and observe real-life activities.
